# Effects of Active Dry Yeast on Production Performance, Meat Quality, and Rumen Microecology in Lambs

**DOI:** 10.3390/ani16081228

**Published:** 2026-04-17

**Authors:** Borui Han, Xuegang Shi, Chen Zheng, Hanfang Zeng, Yi Wang, Ting Liu

**Affiliations:** 1 College of Animal Science and Technology, Gansu Agricultural University, Lanzhou 730070, China; 13007306260@163.com (B.H.); mazh_7853@hotmail.com (C.Z.); 14236@gsau.edu.cn (H.Z.); 2 College of Veterinary Medicine, Gansu Agricultural University, Lanzhou 730070, China; sxg123mx@163.com; 3 Lanzhou Agricultural Radio and Television School, Lanzhou 730030, China; wangyits0505@foxmail.com

**Keywords:** lambs, active dry yeast, production performance, meat quality, rumen microbiota

## Abstract

In this study, we examined whether dietary ADY improves growth, digestibility of feed nutrients, meat quality, and rumen microbial ecology in lambs. This experiment enrolled 90 healthy, similarly weighted (29.0 ± 0.5 kg) four-month-old Duhan lambs, which were randomly and evenly distributed into two treatment groups: a control group fed the basal diet and an ADY group fed the basal diet supplemented with 0.3 g/d per lamb of active dry yeast. The supplementation amount was adjusted weekly according to feed intake to maintain a constant daily dose. The results showed that, compared with the control group, ADY significantly increased the lambs’ average daily gain (ADG) and enhanced the apparent digestibility of neutral detergent fiber (NDF), crude protein (CP) (*p* < 0.05), and significantly reduced the feed conversion ratio (F/G) (*p* < 0.05). These improvements were accompanied by a shift in rumen fermentation toward propionate production, evidenced by higher NH_3_-N, Total volatile fatty acids (TVFAs) and propionate proportion and a lower acetate proportion and acetate-to-propionate ratio (*p* < 0.05). ADY also altered the rumen microbiota, increasing Proteobacteria and *Succinivibrionaceae_UCG-001* while decreasing *norank_o_Clostridia_UCG-014* (*p* < 0.05). In muscle, ADY significantly increased the proportions of C14:0 and C18:3n-3 (*p* < 0.05). In addition, the proportion of C13:0, C18:0 and C18:2n-6t were significantly reduced (*p* < 0.05). In conclusion, dietary supplementation with ADY enhanced rumen fermentation, improved rumen microbial composition, and promoted nutrient utilization in lambs, thereby improving growth performance and meat quality. In addition, certain rumen microbial taxa may be associated with the formation of specific muscle fatty acids.

## Simple Summary

This study investigated how supplementing Active dry yeast (ADY) influences lamb production performance, meat quality, and the rumen microbial ecosystem. ADY appears to optimize rumen fermentation by reshaping the rumen microbiota and shifting fermentation characteristics. In turn, these alterations enhance feed nutrient utilization and may alter muscle fatty acid composition, thereby improving growth performance and selected meat quality traits.

## 1. Introduction

ADY is a microbial feed additive mainly composed of *Saccharomyces cerevisiae* [1]. In broilers, adding ADY to the diet has been reported to enhance growth performance, with increases observed in both ADG and dry matter intake (DMI) [2,3], similar conclusions were reached in pig trials [4,5]. Additionally, in ruminant studies, it has been shown to maintain stable rumen pH, enhance feed fiber degradation rates, and improve rumen microecology [6,7]. Accordingly, the addition of ADY to feed plays a crucial role in promoting ruminant production. For instance, Li et al. [8] reported increased milk yield and milk fat production in Holstein dairy cows, and Ma et al. [9] observed improved ADG and feed efficiency in weaned calves; similar responses have been observed in fattening sheep [10]. Mechanistically, Zhang et al. [11] found that ADY increased *Selenomonas ruminantium*, which can convert intermediates such as lactate and succinate to propionate and acetate [12], potentially mitigating ruminal acidosis and improving energy utilization. Collectively, these findings suggest that ADY may enhance ruminant performance through modulation of rumen microecology.

The rumen microbiota is dominated by bacteria, fungi, and protozoa. These microbes convert dietary substrates into volatile fatty acids (VFAs), including acetate, propionate, and butyrate, which are absorbed through the rumen epithelium and serve as major energy sources for the host [13]. Additionally, rumen microorganisms can promote feed lipid metabolism and influence the composition and content of fatty acids (FAs) within the body. Dietary lipids are hydrolyzed in the rumen, and unsaturated fatty acids (UFA) can be converted to saturated fatty acids (SFA) via biohydrogenation [14] before absorption in the small intestine and deposition in muscle tissue. Feng et al. [15] demonstrated that dietary ADY improved the rumen microbial community of finishing bulls and was associated with changes in ruminal biohydrogenation and UFA content in beef.

As a functional feed additive, ADY has been widely used to improve rumen function and animal productivity; however, the extent to which ADY modulates muscle fatty acid composition through rumen microbial regulation remains unclear. Therefore, this study investigated the effects of ADY supplementation on growth performance, rumen fermentation and microbiota, and meat quality in lambs, providing evidence for the application of ADY in efficient lamb production.

## 2. Materials and Methods

### 2.1. Experimental Materials

The ADY used in this experiment was provided by AB Calsa, S.A. de C.V. (AB Mauri Mexico, Cuitláhuac, Veracruz, Mexico). and had a viable count of ≥2.0 × 10^10^ CFU/g, moisture content ≤ 9.0%, crude ash content ≤ 8.0%, and heavy metal content as follows: total arsenic ≤ 2.0 mg/kg, lead ≤ 5.0 mg/kg, mercury ≤ 0.1 mg/kg, and cadmium ≤ 0.5 mg/kg. The Ethics Committee of the Gansu Agricultural University approved all experimental protocols and sample collections under permit number GSAU-Eth-AST-2024-023.

### 2.2. Experimental Design and Diet Composition

This experiment enrolled 90 healthy, similarly weighted (29.0 ± 0.5 kg) four-month-old Duhan lambs, which were randomly and evenly distributed into two treatment groups. The control group (CON) received the basal diet, whereas the ADY group received the same basal diet supplemented with ADY at 0.3 g/d/lamb. The amount of ADY supplemented was adjusted weekly according to feed intake to maintain a constant daily dose. The basal diet composition and nutrient levels are presented in Table 1.

### 2.3. Experimental Management

The feeding trial lasted 67 days, including a 7-day adaptation (pre-trial) period followed by a 60-day main trial period. During the main trial, animals were fed twice daily at 07:00 and 17:00, with ad libitum access to drinking water. All experiments in this study were conducted following the approved guidelines of the Regulation Standing Committee of the Gansu People’s Congress.

### 2.4. Sample Collection and Indicator Measurement

#### 2.4.1. Growth Performance

Test lambs were weighed in a fasting state before morning feeding. The body weight measured on day 1 of the formal trial was defined as the initial body weight, that measured on day 30 as the intermediate body weight, and that measured on day 60 as the final body weight. ADG for each period was then calculated accordingly. The residual feed was collected daily to calculate DMI.

#### 2.4.2. Nutrient Apparent Digestibility

During days 54–60 of the formal period, five randomly selected lambs per group underwent digestion metabolism trials in metabolic cages. During the trial, feeding adhered to standard protocols. Diet samples were collected, and residual feed and feces were weighed and recorded for each lamb before morning feeding daily. Feces were collected using the total collection method. NDF and ADF content in feed and manure were determined using the Van Soest method [17]. CP and dry matter (DM) content were measured according to AOAC standard methods [18]. Multiple subsamples from each individual lamb were analyzed to improve analytical precision, and the mean value for each lamb was used for statistical analysis. The individual lamb was considered the experimental unit.

#### 2.4.3. Rumen Fermentation Parameters

Before the morning feeding on the final day of the formal trial, rumen fluid was collected from each lamb using an oral stomach tube. After filtering the rumen fluid through four layers of sterile gauze, its pH was measured immediately using a pH meter (PHS-3C, Shanghai INESA Scientific Instrument Co., Ltd., Shanghai, China). Aliquots were transferred into 5 mL cryogenic tubes, snap-frozen in liquid nitrogen for transport, and stored at −80 °C until analysis. After thawing, samples were centrifuged at 5000 rpm, and the supernatant was acidified with metaphosphoric acid to inhibit microbial activity. VFAs were quantified using an Agilent 6890N gas chromatograph (Agilent Technologies, Inc., Santa Clara, CA, USA) equipped with an HP19091N-213 column and an internal standard method. Final VFA concentrations were calculated from peak areas. The concentration of ammonia nitrogen (NH_3_-N) was determined using colorimetric spectrophotometry [19]. The concentration of microbial crude protein (MCP) was determined using the Coomassie Brilliant Blue method [20]. Rumen fluid was collected on the final day of the formal feeding trial; therefore, the fermentation-related measurements in this study represent an end-point observation rather than dynamic changes across the entire feeding period.

#### 2.4.4. Rumen Microbiota

The collected rumen fluid samples were sent to Shanghai Majorbio Bio-Pharm Technology Co., Ltd. (Shanghai, China) for 16S rRNA sequencing. Total genomic DNA from rumen microbial communities was extracted using the E. Z. N. A.^®^ soil DNA kit (Omega Bio-tek, Norcross, GA, USA). After quality and concentration assessment by 1% agarose gel electrophoresis and NanoDrop 2000 (Thermo Fisher Scientific, Waltham, MA, USA), the extracted DNA was used as the template for PCR amplification of the V3-V4 region of the 16S rRNA gene using the barcoded primers 338F (5′-ACTCCTACGGGAGGCAGCAG-3′) and 806R (5′-GGACTACHVGGGTWTCTAAT-3′). Amplified products were purified from 2% agarose gel, quantified, and then library-prepared using the NEXTFLEX Rapid DNA-Seq Kit (Thermo Fisher Scientific, Waltham, MA, USA). Sequencing was performed on an Illumina NextSeq 2000 platform. Data quality control and assembly were performed using fastp (v0.19.6)and FLASH (v1.2.11). Operational taxonomic units (OTUs) were clustered at 97% similarity using UPARSE, chimeras were removed, and sequences were normalized before taxonomic annotation against the SILVA database using the RDP classifier. We adopted an OTU-based workflow because UPARSE remains a widely used and well-established de novo clustering method for 16S rRNA amplicon data, and this study focuses primarily on community-level diversity patterns and shifts in dominant taxa rather than single-nucleotide sequence variation. Rumen fluid was collected on the final day of the formal feeding trial; therefore, the microbiota data in this study represent an end-point observation rather than dynamic changes across the entire feeding period.

#### 2.4.5. Meat Quality

At the end of the formal trial (day 60), 12 lambs with good body condition and body weights close to the treatment mean were randomly selected from each group. The selected lambs were fasted for 12 h with free access to water according to routine pre-slaughter procedures, weighed the following morning before slaughter, and then transported to the abattoir for processing. After slaughter, the longissimus dorsi muscle was collected from experimental lambs. Muscle pH was measured at 45 min and 24 h post-slaughter using the same pH meter (PHS-3C, Shanghai INESA Scientific Instrument Co., Ltd., Shanghai, China) fitted with a spear-type penetration electrode suitable for semi-solid samples. Muscle L*, a*, and b* values were determined using a Chroma Meter CR-400/410 colorimeter (Konica Minolta, Inc., Tokyo, Japan). Drip loss, cooking loss, and water loss rate were determined according to the method of Honikel [21].

#### 2.4.6. Muscle Fatty Acids

Quantitative analysis of longissimus dorsi (LD) muscle fatty acid composition was performed using a gas chromatography method established by O’Fallon et al. [22]. LD muscle samples were thawed at 4 °C, and surface fat and fascia were removed. Samples were frozen in liquid nitrogen and ground in a mortar. An aliquot (1.0 g) of homogenized tissue was weighed into a 15 mL centrifuge tube, and 0.7 mL of 10 N potassium hydroxide solution followed by 5.3 mL of methanol were added. Tubes were incubated in a 55 °C water bath for 1.5 h, mixing every 20 min. After cooling to room temperature, 0.58 mL of 24 N sulfuric acid solution was added, mixed thoroughly, and the incubation step was repeated. After cooling, 1 mL of hexane was added and vortexed thoroughly. The mixture was centrifuged at 5000 rpm for 10 min, and the supernatant was filtered through a 0.22 μm membrane filter into amber GC vials for chromatographic analysis (Agilent 6890N, Agilent Technologies, Inc., Santa Clara, CA, USA). Individual FAs were quantified based on peak area and expressed as a percentage of total FAs.

### 2.5. Data Analysis

Growth performance data were analyzed using R software (v3.5.1). Because BW was repeatedly measured on d 1, 30, and 60 of the formal trial, BW was analyzed using a linear mixed-effects model with treatment, day, and their interaction as fixed effects, and lamb as a random effect to account for repeated measurements within the same animal. The model was as follows:

$$Y_{ijk}=\mu +T_{i}+D_{j}+{\left(T\times D\right)}_{ij}+L_{k}+\epsilon_{ijk}$$

where Y_ijk_ is the BW observation of lamb k under treatment i on day j; μ is the overall mean; T_i_ is the fixed effect of treatment (i = 1, 2; CON and ADY); D_j_ is the fixed effect of day (j = 1, 2, 3; d 1, d 30, and d 60); (T × D)_ij_ is the fixed effect of the treatment × day interaction; L_k_ is the random effect of lamb; and ϵ_ijk_ is the residual error.

ADG, DMI, and F/G for the two production phases (d 0–30 and d 31–60) were also analyzed using a linear mixed-effects model with treatment, phase, and their interaction as fixed effects, and lamb as a random effect:

$$Y_{ijk}=\mu +T_{i}+P_{j}+{\left(T\times P\right)}_{ij}+L_{k}+\epsilon_{ijk}$$

where Y_ijk_ is the observed value of ADG, DMI, or F/G of lamb k under treatment i during phase j; P_j_ is the fixed effect of phase (j = 1, 2; d 0–30 and d 31–60); and the remaining terms are as defined above.

For variables measured at a single time point, including apparent nutrient digestibility, rumen fermentation parameters, meat quality traits, and muscle fatty acid composition, differences between the CON and ADY groups were evaluated using an independent-samples *t*-test. These statistical analyses were performed using SPSS 28.0 (SPSS Inc., Chicago, IL, USA). The model used was as follows:

$$t=\frac{{\bar{x}}_{1}-{\bar{x}}_{2}}{S_{{\bar{x}}_{1}-{\bar{x}}_{2}}}$$

where ${\bar{x}}_{1}$ and ${\bar{x}}_{2}$ are the means of different treatments, and $S_{{\bar{x}}_{1}-{\bar{x}}_{2}}$ is the standard error of the difference between the means.

For α diversity analysis, we employed the R package Phyloseq, and for β analysis, we utilized the R package Vegan. Spearman’s rank correlation analysis was used to assess associations between microbial taxa and rumen fermentation parameters as well as muscle fatty acid profiles. Statistical significance was defined as *p* < 0.05, and correlations with |r| > 0.5 were considered biologically meaningful.

## 3. Results

### 3.1. Effects of Active Dry Yeast on Growth Performance of Lambs

As shown in Table 2, regarding body weight, the main effect of treatment was not significant (*p* > 0.05), the main effect of time was significant (*p* < 0.05), and the interaction between treatment and time was not significant (*p* > 0.05). Regarding average daily weight gain, the ADY group was significantly higher than the CON group (*p* < 0.05); the main effect of time was significant (*p* < 0.05); and the interaction between treatment and time was not significant (*p* > 0.05). Regarding DMI, the main effect of treatment was not significant (*p* > 0.05), the main effect of time was significant (*p* < 0.05), and the interaction between treatment and time was not significant (*p* > 0.05). Regarding F/G, the ADY group was significantly lower than the CON group (*p* < 0.05), the main effect of time was significant (*p* < 0.05), and the interaction between treatment and time was not significant (*p* > 0.05).

### 3.2. Effects of ADY on the Apparent Digestibility of Nutrients in Lambs

As shown in Table 3, the addition of ADY significantly increased the apparent digestibility of CP and NDF in the experimental lambs (*p* < 0.05), while no significant difference was observed in the apparent digestibility of DM and ADF (*p* > 0.05).

### 3.3. Effects of Active Dry Yeast on Rumen Fermentation Parameters in Lambs

As shown in Table 4, the addition of ADY significantly increased (*p* < 0.05) NH_3_-N, TVFAs, and the molar proportion of propionate, while decreasing (*p* < 0.05) the molar proportion of acetate and the acetate-to-propionate (A/P) ratio. Rumen pH and the molar proportions of other VFAs did not differ between treatments (*p* > 0.05).

### 3.4. Effects of Active Dry Yeast on the Composition of Lamb Rumen Microbiota

#### 3.4.1. OTU Analysis of Lamb Rumen Microorganisms

**Figure 1:**
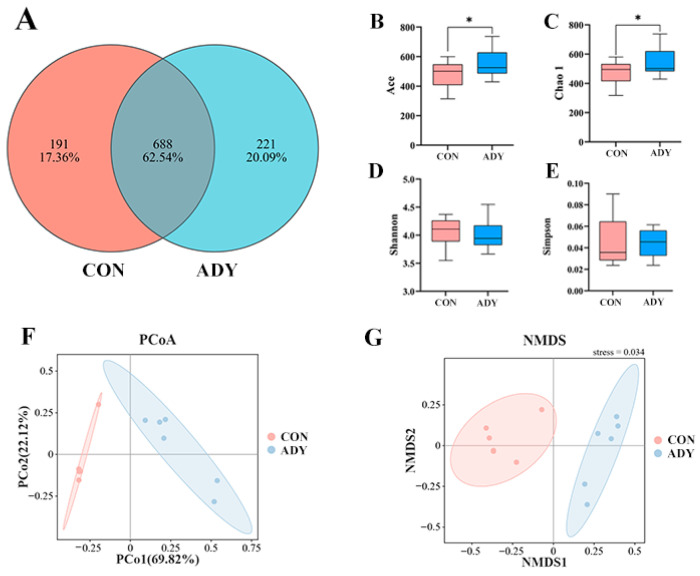
Effect of Active Dry Yeast on alpha- and beta-diversity analyses in the Rumen of Lambs. (**A**) Venn diagram. The OTUs were clustered effectively based on 97% similarity. (**B**–**E**) ACE, Chao 1, Shannon and Simpson. (**F**,**G**) PCoA, NMDS. Microbial community structure was visualized using PCoA (Principal Coordinate Analysis) based on Bray–Curtis distances and NMDS. CON means control group, ADY means Active dry yeast group. The same applies to the following figure. * indicates a significant difference between the two groups.

As shown in Figure 1A, OTUs were effectively clustered based on 97% similarity, yielding a total of 1100 OTUs across both groups. There are 688 identical OTUs between the two groups. The CON group contained 191 unique OTUs, while the ADY group identified 221 unique OTUs.

#### 3.4.2. Alpha Diversity, Principal Coordinates, and Non-Metric Multidimensional Scaling Analysis of Lamb Rumen Microorganisms

As shown in Figure 1, alpha-diversity analysis (Figure 1B–E) indicated that the Chao1 and ACE indices were significantly higher in the ADY group than in the CON group (*p* < 0.05). PCoA and NMDS based on Bray–Curtis distances indicated differences in overall community structure between the CON and ADY groups (Figure 1F,G), suggesting that ADY supplementation increased community richness and altered overall community structure.

#### 3.4.3. Analysis of Ruminal Microbial Composition

**Figure 2:**
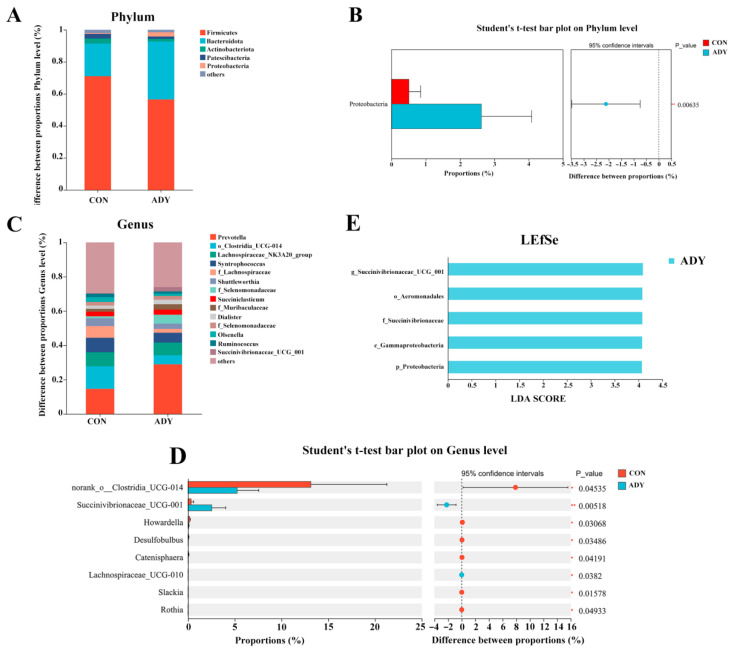
Effect of Active Dry Yeast on Microbial Diversity in the Rumen of Lambs. (**A**,**C**) Ruminal microbial abundance at phylum and genus levels. (**B**,**D**) Differential taxa at the phylum and genus levels. (**E**) Analysis of differences in LEfSe of ruminal microorganisms. The magnitude of the LDA horizontal axis values reflects the degree to which microbial taxa contribute to the differences between groups (LDA value ≥ 4). * and **, *p* < 0.05.

As shown in Figure 2, the microbial composition results at the phylum level (Figure 2A) and genus level (Figure 2C) for the two rumen fluid groups are presented. The top five phyla by relative abundance were Firmicutes, Bacteroidetes, Actinobacteria, Patescibacteria, and Proteobacteria. Notably, the relative abundance of Proteobacteria in the ADY group was significantly higher than that in the CON group (*p* < 0.05) (Figure 2B). At the genus level, the top five genera by relative abundance were *Prevotella*, *norank_o_Clostridia_UCG-014*, *Lachnospiraceae_NK3A20_group*, *Syntrophococcus*, and *unclassified_f_Lachnospiraceae* (Figure 2C). Notably, the relative abundance of *norank_o_Clostridia_UCG-014* in the rumen of the CON group was significantly higher than that in the ADY group (*p* < 0.05), while the relative abundance of *Succinivibrionaceae_UCG-001* was significantly lower than that in the ADY group (*p* < 0.05) (Figure 2D).

#### 3.4.4. Linear Discriminant Analysis (LDA) Effect Size Analysis (LEfSe) of Lamb Rumen Microbiota

As shown in Figure 2E, we employed LEfSe analysis to search for biomarkers from phylum to genus levels (LDA ≥ 4.0). The results revealed five significantly enriched dominant bacterial communities in the ADY group, primarily including *g_Succinivibrionaceae_UCG-001*, *o_Aeromonadales*, *f_Succinivibrionaceae, c_Gammaproteobacteria,* and *p_Proteobacteria*. It is noteworthy that no significantly enriched dominant bacterial communities were observed in the CON group.

### 3.5. Effects of Active Dry Yeast on Lamb Meat

As shown in Table 5, ADY supplementation significantly reduced (*p* < 0.05) the pH of the longissimus dorsi at 45 min postmortem, decreased muscle yellowness (b*) and drip loss. In contrast, brightness (L*) increased (*p* < 0.05). Other meat quality traits did not differ between treatments (*p* > 0.05).

### 3.6. Effects of Active Dry Yeast on Fatty Acids Profiles in Lamb Muscle

As shown in Table 6, ADY supplementation significantly increased (*p* < 0.05) the proportions of C14:0 and C18:3n-3 in the longissimus dorsi muscle. In addition, the proportions of C13:0, C18:0 and C18:2n-6t were significantly reduced (*p* < 0.05). No significant differences were observed in the proportions of the other measured FAs (*p* > 0.05).

### 3.7. Correlation Analysis of Lamb Rumen Microorganisms and Muscle Fatty Acids

**Figure 3:**
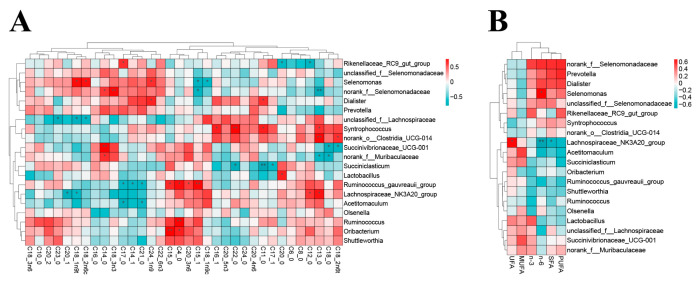
Correlation analysis of rumen microorganisms and muscle fatty acids in lambs. (**A**) Correlation analysis between individual fatty acids and rumen microorganisms. (**B**) Correlation analysis between fatty acid groups and rumen microorganisms. The color shade is proportional to the correlation value. * and ** |r| > 0.5, *p* < 0.05.

As shown in Figure 3, Spearman’s correlation analysis identified 53 significant associations between the top 20 dominant ruminal genera and muscle FAs. For clarity, only the associations with greater biological relevance are presented here. Within the ADY-associated microbial shifts and the fatty acid correlation network, *Succinivibrionaceae_UCG-001* was significantly positively correlated with C14:0, whereas it was significantly negatively correlated with C18:0 and C18:2n-6t. *Selenomonas* showed negative correlations with C18:1n-9c and C15:1, but positive correlations with C24:1n-9, C18:2n-6c, C18:1n-9t, and PUFA n-6. In addition, *Lachnospiraceae_NK3A20_group* was negatively correlated with C20:1, C18:1n-9t, PUFA n-6, and SFA, while showing positive correlations with C12:0, C13:0, and UFA. Several genera were also significantly associated with odd-chain fatty acids or microbially derived fatty acids. Specifically, *Ruminococcus_gauvreauii_group* was negatively correlated with C14:1, C17:0, and C21:0, but positively correlated with C4:0, C15:0, C15:1, and C20:3n-6, whereas *Acetitomaculum* was negatively correlated with C17:0 and C21:0.

### 3.8. Correlation Analysis of Rumen Microorganisms and Rumen Volatile Fatty Acids in Lambs

**Figure 4:**
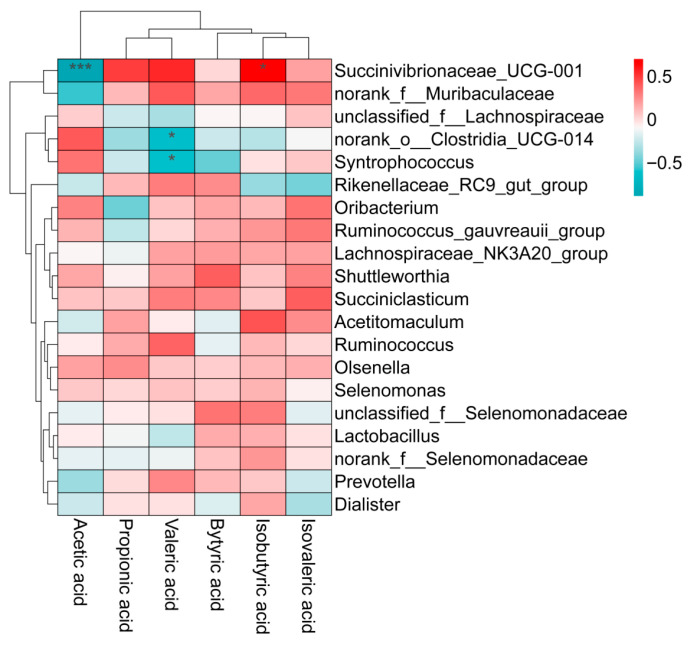
Correlation analysis between rumen microbiota and ruminal volatile fatty acids in lambs. The color shade is proportional to the correlation value. * and *** |r| > 0.5, *p* < 0.05.

As shown in Figure 4, the correlation heatmap based on Spearman’s rank correlation analysis revealed four significantly correlated (“bacteria–volatile fatty acid”) pairs among the top 20 dominant rumen bacterial genera by relative abundance and rumen VFAs. Specifically, *Succinivibrionaceae_UCG-001* showed a significant positive correlation with isobutyrate and a significant negative correlation with acetate. *norank_o_Clostridia_UCG-014* showed a significant negative correlation with valerate. *Syntrophococcus* exhibited a significant negative correlation with valerate.

## 4. Discussion

### 4.1. Effects of Active Dry Yeast on Growth Performance in Lambs

Growth performance serves as a crucial indicator for evaluating animal growth status. In the present study, ADY supplementation increased ADG from 371.27 to 413.33 g/d during d 0–30 and from 282.06 to 301.39 g/d during d 30–60, while reducing F/G from 5.20 to 4.80 and from 7.32 to 6.86, respectively, with no significant effect on DMI. These results suggest that the growth-promoting effect of ADY in the current study was primarily associated with improved efficiency of feed utilization rather than increased feed intake. Time significantly affected BW, ADG, DMI, and F/G, reflecting the normal growth process of lambs and the dynamic changes in nutrient utilization during the experiment. Similar results have been reported in weaned beef calves and fattening sheep, where ADY improved ADG, feed efficiency, and overall production performance [9,10]. Liu et al. [23] also observed improved growth performance in beef cattle supplemented with dry yeast, whereas Zhang et al. [24] reported increases in both DMI and ADG in dairy goats. Burt et al. [25] further confirmed the positive effect of *Saccharomyces cerevisiae* fermentation products on lamb feedlot performance. Therefore, ADY can improve growth performance in lambs mainly by enhancing nutrient utilization efficiency.

### 4.2. Effect of Active Dry Yeast on Nutrient Apparent Digestibility in Lambs

Nutrient Apparent Digestibility reflects the extent of feed digestion in animals and serves as a crucial indicator for evaluating feed absorption [17]. For ruminants, nutrient digestibility primarily depends on rumen microbial communities—including fiber-degrading bacteria, starch-degrading bacteria, and protein-degrading bacteria—which enhance feed digestion and absorption. These microbes also break down nutrients indigestible by the animal, converting them into smaller molecules for absorption and improving feed utilization. In the present study, ADY supplementation increased CP digestibility from 59.59% to 66.96% and NDF digestibility from 57.29% to 61.99%, whereas DM digestibility and ADF digestibility were not significantly affected. These results indicate that ADY improved the utilization of dietary protein and cell wall fractions, which is consistent with the study of Obeidat et al. [26], who also reported that *Saccharomyces cerevisiae* supplementation improved nutrient digestibility in lambs. The increased digestibility of CP and NDF indicates that ADY enhances the utilization of nutrients in the rumen. However, the sequencing results from this study did not reveal a significant enrichment of fiber-degrading bacteria in the ADY group. Therefore, the improvements in these parameters may be attributed to enhanced rumen fermentation efficiency, substrate colonization capacity, and the activity of fiber-degrading bacterial communities following the addition of ADY. Nevertheless, further validation is still required in future studies with larger sample sizes.

### 4.3. Effects of Active Dry Yeast on Rumen Fermentation Parameters in Lambs

VFAs are metabolic products of rumen microorganisms in ruminants and serve as an important energy source for animals [13]. In this study, the addition of ADY significantly increased TVFA levels (from 67.38 to 79.46 mmol/L) in the rumen, indicating enhanced fermentation of the diet within the rumen and providing the host with more energy-yielding substrates [27]. Concurrently, this was accompanied by an increase in the molar ratio of propionic acid (from 30.24 to 39.97%) and a decrease in both the molar ratio of acetic acid (from 62.44 to 52.16%) and the A/P ratio (from 2.19 to 1.31). This signifies a shift in rumen carbon flux from acetogenic to propionic acid production pathways, and a reduced A/P ratio is often associated with improved feed conversion efficiency [28]. In this study, *Succinivibrionaceae_UCG-001* showed a significant negative correlation with acetate, and its relative abundance significantly increased in the ADY group, consistent with the decreasing trend of the acetate molar ratio. This indicates that ADY promotes a shift toward a propionate-producing fermentation pattern by enriching this bacterial group, corroborating the previous findings. Both acetate and propionate are energy-related VFAs in the rumen. However, acetate-producing pathways generate hydrogen, while propionate-producing pathways consume hydrogen [29]. Therefore, increasing the propionate proportion reduces energy loss due to methane production, making rumen fermentation more energy-efficient. Additionally, propionic acid serves as the primary precursor for glucose synthesis in ruminants. It is converted into glucose via hepatic gluconeogenesis, supplying energy to the body [30,31]. Compared to acetate, propionic acid offers a more direct energy pathway, and its increased proportion promotes better utilization of feed energy.

Rumen pH is a key indicator for evaluating microbial fermentation efficiency and rumen health status in ruminants [32]. In this study, the rumen fluid pH in the ADY group showed no significant change. It should be noted that the basal diet was a concentrate-based finishing diet composed primarily of grains and by-products. It did not include traditional roughage sources such as hay or silage, but peanut shells were added as a fiber source. Under these feeding conditions, the supply of easily fermentable carbohydrates may increase the risk of postprandial rumen pH decline and fermentation instability. In this study, although rumen pH measured at slaughter did not change significantly with the addition of ADY, ADY increased the molar ratios of TVFAs and propionic acid, while reducing the acetic acid ratio and the acetic acid/propionic acid ratio, without a concomitant decrease in rumen pH. This pattern suggests that, under this concentrated diet, ADY helps maintain rumen stability while improving fermentation efficiency. This suggests that the use of ADY under a high-concentrate diet is of significant importance.

ADY supplementation significantly increased ruminal NH_3_-N concentration (from 6.37 to 6.72 mg/dL), whereas MCP did not increase significantly. Ruminal NH_3_-N reflects the balance between ammonia release from degradable nitrogen sources and its capture by rumen microorganisms [33]. Therefore, the increase in NH_3_-N may indicate that ADY promoted ruminal fermentation activity and accelerated the degradation of dietary protein and other nitrogenous substrates, thereby increasing ammonia availability in the rumen [34]. Previous studies have also shown that the effect of yeast supplementation on ruminal ammonia is influenced by dietary conditions [35]. In contrast, the lack of a significant increase in MCP suggests that the additional ammonia was not fully converted into microbial protein. This may be because microbial protein synthesis depends not only on nitrogen supply, but also on the availability of fermentable energy and the synchrony between energy and nitrogen release in the rumen [36]. Thus, although ADY increased ruminal nitrogen turnover, the conditions for microbial protein synthesis may not have been sufficiently optimized to produce a significant rise in MCP.

In summary, ADY increases TVFA while maintaining rumen pH levels, elevates propionic acid proportion, reduces acetic acid proportion and A/P ratio, and promotes a shift toward more efficient propionic acid-producing fermentation. This change may contribute to promoting protein degradation in feed and enhancing feed utilization efficiency.

### 4.4. Effects of Active Dry Yeast on Rumen Microbial Community Composition in Lambs

The stability and diversity of rumen microbial communities are closely linked to rumen fermentation homeostasis and host nutrient utilization efficiency [37]. At the phylum level, the dominant rumen microbial phyla in both groups were primarily Firmicutes, Bacteroidetes, Actinobacteria, Patescibacteria, and Proteobacteria, consistent with the fundamental characteristic of rumen microbiota in ruminants being dominated by polysaccharide and protein-fermenting bacteria [38]. The relative abundance of Proteobacteria was significantly higher in the ADY group than in the CON group. Based on rumen fermentation parameters, this increase may be associated with the elevated abundance of energy metabolism-related Proteobacteria. At the genus level, *Prevotella* exhibited the highest relative abundance in both groups, though the difference was not significant. This may be because *Prevotella* is a core rumen genus with strong substrate adaptability, capable of degrading various carbon sources (especially non-structural carbohydrates and certain proteins/peptides) [39]. Thus, its abundance remained relatively stable across groups. Notably, the relative abundance of *norank_o_Clostridia_UCG-014* was significantly higher in the CON group than in the ADY group, while that of *Succinivibrionaceae_UCG-001* was significantly higher in the ADY group than in the CON group. This suggests that ADY may alter the rumen microenvironment and substrate flow [40,41], promoting a shift in the rumen microbial community from partially inefficient energy-acquiring groups toward those with higher energy metabolism and fermentation efficiency. Particularly, the increase in *Succinivibrionaceae_UCG-001* aligns directionally with the earlier observed rise in propionic acid molar ratio and decrease in A/P ratio, suggesting that the dominant microbial communities induced by ADY are more likely associated with enhanced propionic acid fermentation pathways, thereby improving feed energy recovery efficiency. This finding was further validated by LEFSe analysis. At the LDA ≥ 4.0, biomarkers significantly enriched in the ADY group primarily included *g_Succinivibrionaceae_UCG-001*, *o_Aeromonadales*, *f_Succinivibrionaceae*, *c_Gammaproteobacteria*, and *p_Proteobacteria*, whereas no distinct dominant microbial groups were detected in the CON group. This indicates that ADY’s effect on rumen microbiota primarily promotes groups associated with energy supply and fermentation efficiency. This indicates that the core function of the rumen microbiota remains primarily focused on breaking down dietary nutrients and producing energy substrates [38]; whereas the community structure differences induced by ADY manifest as a redistribution of these fundamental metabolic functions across the microbiota. Nevertheless, further confirmation of the effects on relevant key pathways (such as propionic acid production-related pathways and hydrogen utilization-related pathways) is still needed and requires validation using metagenomic data. In addition, while the preceding text noted an increase in fiber digestibility, no significant enrichment of fiber-degrading bacteria was observed. This may be because fiber-degrading bacteria are typically associated with particulate matter and are therefore likely underestimated in filtered rumen fluid samples compared to mixed or solid rumen contents. In summary, ADY enhances rumen microbial diversity and modulates microbial community structure, directing enrichment of energy-efficient metabolic communities represented by *Succinivibrionaceae_UCG-001*. This provides microbiological support for shifting fermentation patterns toward propionic acid production and enhancing feed energy utilization. However, because the microbiota analysis was based on 97% OTU clustering rather than an ASV-based denoising approach, these findings should be interpreted as community-level shifts rather than fine-scale sequence variant resolution.

### 4.5. Effects of Active Dry Yeast on Lamb Meat Quality

Muscle pH is a key indicator of postmortem glycolysis and meat quality [42]. After slaughter, muscle glycogen is converted to lactic acid, and rapid glycolysis can accelerate pH decline [43]. In the present study, pH45 min was significantly lower in the ADY group (5.86) than in the CON group (6.11), and pH45 min was slightly below the conventional range (pH45 min: 6.0–6.8; pH24 h: 5.3–5.8) [44]. This may be associated with the high molar ratio of propionic acid observed in the ADY group, which promotes early glycolysis and accelerates lactate formation. Thereby promoting a faster pH decline at 45 min. Nevertheless, the above inference suggests only a possible association; the specific mechanisms require further research and observation. However, no significant differences were observed in water loss rate, cooking loss, or shear force, and the ultimate pH at 24 h remained within the normal range in both groups. These results suggest that ADY supplementation may have accelerated early postmortem acidification without causing clear deterioration in ultimate water-holding capacity or tenderness under the present experimental conditions. In contrast, ADY supplementation reduced drip loss (from 4.96 to 4.08%), suggesting an improvement in the ability of the muscle to retain water after slaughter. Water-holding capacity is closely related to postmortem changes in muscle proteins and structure [43]. The lower drip loss observed in the ADY group may indicate that ADY contributed to maintaining better muscle water retention, thereby improving meat quality [45]. Therefore, the observed reduction in pH45 min is better interpreted as an alteration in the early rate of pH decline rather than definitive evidence of a meat quality defect. Concurrently, the increase in L* (from 22.76 to 26.06) and decrease in b* (from 5.93 to 4.20) shifted the overall muscle color toward the brighter red hue preferred by consumers. This may be associated with ADY-induced changes in muscle fatty acid composition; however, because lipid oxidation indicators were not measured in the present study, the mechanism underlying the color change remains to be further clarified. Overall, dietary ADY supplementation appeared to alter the rate of early post-mortem pH decline in lamb meat without adversely affecting meat quality; instead, it contributed to improvements in certain meat quality traits.

### 4.6. Effects of Active Dry Yeast on Fatty Acid Profiles in Lamb Muscle

In this study, ADY increased C14:0 and C18:3n-3, while decreasing C13:0, C18:0, and C18:2n-6t. When these results were considered together with the correlation analysis, it seems likely that ADY influenced muscle fatty acid composition mainly by changing rumen microbial activity and lipid metabolism, rather than simply increasing fat deposition. Notably, *Succinivibrionaceae_UCG-001*, which was more abundant in the ADY group, was positively correlated with C14:0 but negatively correlated with C18:0 and C18:2n-6t. In contrast, *Lachnospiraceae_NK3A20_group* was positively correlated with C13:0. These relationships suggest that changes in the rumen microbiota may have contributed to the changes in specific fatty acids found in muscle.

The increase in C14:0 may be related to ADY-induced changes in rumen fermentation and microbial metabolism, which could have influenced fatty acid synthesis and deposition in muscle. In the present study, *Succinivibrionaceae_UCG-001* was positively correlated with C14:0, suggesting that shifts in the rumen microbial community may have contributed to the higher C14:0 proportion observed in the ADY group. The decrease in C13:0 may be linked to changes in microbial-derived fatty acids. Odd-chain and branched-chain fatty acids are often regarded as indicators of rumen microbial lipids, because many rumen bacteria contain relatively high amounts of these FAs [46,47]. Therefore, the lower C13:0 level in the ADY group may reflect changes in the abundance or activity of certain microbial populations. The positive correlation between *Lachnospiraceae_NK3A20_group* and C13:0 is in line with this possibility.

The decreases in C18:0 and C18:2n-6t may be associated with changes in ruminal biohydrogenation. In the rumen, microorganisms convert unsaturated C18 fatty acids into trans intermediates and finally into more saturated products such as C18:0. Therefore, C18:0 is commonly considered an end product of biohydrogenation, whereas C18:2n-6t represents an intermediate in this process [47,48,49]. The simultaneous decrease in both C18:0 and C18:2n-6t suggests that ADY may have affected the pathway or extent of ruminal biohydrogenation. This idea is also supported by the negative correlations of *Succinivibrionaceae_UCG-001* with these two FAs. The higher C18:3n-3 content may indicate better retention of n-3 fatty acids in muscle. Alpha-linolenic acid is highly susceptible to biohydrogenation in the rumen, and previous studies have shown that when its biohydrogenation is reduced, more of it can pass to the intestine and eventually be deposited in tissues [50,51]. Thus, the increase in C18:3n-3 observed here may suggest that ADY helped preserve part of this fatty acid during rumen metabolism. This is also consistent with the decreases in C18:0 and C18:2n-6t.

Overall, ADY may have influenced the fatty acid composition of lamb muscle by changing rumen microbial structure and biohydrogenation-related lipid metabolism. It was manifested as ADY supplementation did not markedly change the total FAs in lamb muscle, but selectively modified the levels of some specific FAs.

## 5. Conclusions

Under the experimental conditions, ADY enhanced rumen fermentation and modified selected muscle fatty acids, while improving nutrient utilization and growth performance in lambs.

## Figures and Tables

**Table 1:** Basic diet ingredients and nutrient composition (as-fed basis).

Items	Content
Ingredients (%)	
Corn	23.60
Corn Germ Meal	20.00
Sprayed corn bran	15.00
Corn flakes	17.00
Peanut shell	10.90
Bran	4.20
Limestone	2.50
Cottonseed meal	2.50
Cane molasses	2.00
Extruded urea ^a^	0.90
Sodium chloride (NaCl)	0.70
Ammonium chloride (NH_4_Cl)	0.40
A dditive premixes ^b^	0.30
Total	100.00
Nutritional compositions ^c^ (%)	
Digestible Energy DE/(MJ·kg^−1^)	11.26
Metabolizable Energy ME/(MJ·kg^−1^)	9.28
DM	92.60
CP	15.00
Crude fiber CF	12.60
NDF	44.09
Acid detergent fiber ADF	15.06
Starch	25.30
Lysine Lys	0.42
Calcium	1.10
Phosphorus	0.45

^a^ Extruded urea is a feed-grade non-protein nitrogen source used to supply ammonia for rumen microbial growth and microbial protein synthesis. ^b^ The Additive premix provides per kilogram of diet: Fe 25 mg, Mn 45 mg, Zn 40 mg, Cu 10 mg, I 0.5 mg, Se 0.5 mg, Co 0.2 mg, S 200 mg, VA 2000 IU, VE 50 IU. ^c^ DE and ME are a calculated values [16], while the others were measured values.

**Table 2:** Effects of active dry yeast on growth performance of lambs.

Items	Treatment ^a^		SEM ^b^	*p*-Value		
	CON	ADY		Treat	Time	Treat × Time
BW, kg						
Initial	29.03	29.03	2.96	0.062	<0.001	0.376
Mid-term	40.17	41.43	3.38			
Final	48.63	50.47	3.26			
ADG, g/d						
d 0–30	371.27	413.33	67.29	0.025	<0.001	0.401
d 30–60	282.06	301.39	62.93			
DMI, kg/d						
d 0–30	1.87	1.93	0.09	0.183	<0.001	0.941
d 30–60	2.15	2.21	0.05			
F/G						
d 0–30	5.20	4.80	0.90	0.049	<0.001	0.888
d 30–60	7.32	6.86	1.03			

^a^ CON means control group, ADY means Active dry yeast group. ^b^ SEM means standard error. The same applies to the following table.

**Table 3:** Effects of active dry yeast on apparent nutrient digestibility in lambs.

Items	Treatment		SEM	*p*-Value
	CON	ADY		
DM apparent digestibility (%)	62.94	63.28	1.22	0.784
CP apparent digestibility (%)	59.59	66.96	0.82	<0.001
NDF apparent digestibility (%)	57.29	61.99	0.97	0.001
ADF apparent digestibility (%)	40.25	44.81	3.93	0.279

**Table 4:** Effects of active dry yeast on rumen fermentation parameters in lambs.

Items	Treatment		SEM	*p*-Value
	CON	ADY		
pH	6.44	6.42	0.08	0.760
NH_3_-N (mg/dL)	6.37	6.72	0.09	<0.001
MCP (g/dL)	3.04	3.18	0.12	0.229
TVFAs (mmol/L)	67.38	79.46	3.13	0.003
Acetate (%)	62.44	52.16	2.21	<0.001
Propionate (%)	30.24	39.97	2.35	0.001
Isobutyrate (%)	0.78	1.10	0.18	0.097
Butyrate (%)	4.72	4.10	0.73	0.402
Isovalerate (%)	0.96	1.32	0.29	0.225
Valerate (%)	0.86	1.36	0.24	0.056
A/P	2.19	1.31	0.20	0.001

**Table 5:** Effects of active dry yeast on lamb meat.

Items	Treatment		SEM	*p*-Value
	CON	ADY		
Meat pH				
pH _45 min_	6.11	5.86	0.06	<0.001
pH _24 h_	5.32	5.31	0.02	0.460
Color ^a^				
L*	22.76	26.06	1.06	0.004
a*	16.27	17.59	0.77	0.098
b*	5.93	4.20	0.40	<0.001
Drip Loss/%	4.96	4.08	0.23	0.001
Water Loss Rate/%	29.94	32.18	1.53	0.165
Cooking Loss Rate/%	18.32	19.81	1.85	0.433
Shear force/N	80.07	79.34	2.57	0.777

^a^ Color: L*, brightness of color; a*, redness; b*, yellowness.

**Table 6:** Effects of active dry yeast on muscle fatty acids in lambs (%).

Items	Treatment		SEM	*p*-Value
	CON	ADY		
Butyric acid (C4:0)	0.09	0.11	0.02	0.424
Caproic acid (C6:0)	0.08	0.10	0.01	0.093
Caprylic acid (C8:0)	0.05	0.05	0.01	0.996
Capric acid (C10:0)	0.09	0.12	0.01	0.078
Undecanoic acid (C11:0)	0.06	0.05	0.01	0.436
Lauric acid (C12:0)	0.09	0.08	0.01	0.412
Tridecanoic acid (C13:0)	0.12	0.07	0.02	0.015
Myristic acid (C14:0)	2.08	2.73	0.17	<0.001
Tetradecenoic acid (C14:1)	0.27	0.29	0.07	0.754
Pentadecanoic acid (C15:0)	0.17	0.21	0.04	0.328
Pentadecenoic acid (C15:1)	0.15	0.22	0.05	0.131
Palmitic acid (C16:0)	23.83	25.19	0.69	0.057
Palmitoleic acid (C16:1)	0.34	0.29	0.05	0.308
Heptadecanoic acid (C17:0)	1.97	1.93	0.25	0.860
Heptadecenoic acid (C17:1)	0.86	0.63	0.13	0.082
Stearic acid (C18:0)	12.46	10.52	0.59	0.003
Elaidic acid (C18:1n-9t)	7.08	6.93	0.99	0.877
Oleic acid (C18:1n-9c)	35.77	37.15	1.53	0.373
Trans-linoleic acid (C18:2n-6t)	0.50	0.23	0.12	0.035
Linoleic acid (C18:2n-6c)	7.35	6.86	0.63	0.451
Arachidic acid (C20:0)	0.09	0.09	0.01	0.805
Gamma-Linolenic Acid (C18:3n-6)	0.17	0.20	0.03	0.297
Eicosenoic acid (C20:1)	0.52	0.55	0.09	0.689
α-Linolenic acid (C18:3n-3)	0.17	0.26	0.02	<0.001
Heneicosanoic acid (C21:0)	0.12	0.11	0.02	0.905
Eicosadienoic acid (C20:2)	0.13	0.13	0.02	0.773
Docosanoic acid (C22:0)	0.27	0.25	0.04	0.605
Eicosatrienoic acid (C20:3n-6)	0.17	0.21	0.03	0.175
Tricosanoic acid (C23:0)	0.16	0.10	0.04	0.258
Arachidonic acid (C20:4n-6)	2.36	2.52	0.70	0.827
Tetracosadienoic acid (C24:0)	0.06	0.06	0.01	0.591
Eicosapentaenoic acid (C20:5n-3)	0.51	0.38	0.07	0.051
Nervonic acid (C24:1n-9)	0.22	0.23	0.02	0.463
Docosahexaenoic acid (C22:6n-3)	1.67	1.88	0.15	0.181
SFA ^a^	41.88	41.97	0.62	0.893
UFA ^b^	58.16	58.15	0.72	0.982
Monounsaturated fatty acids, MUFA ^c^	45.27	46.14	0.51	0.100
Polyunsaturated fatty acids, PUFA ^d^	13.05	12.69	0.72	0.611
PUFA n-3 ^e^	2.64	2.64	0.13	0.990
PUFA n-6 ^f^	10.01	9.35	0.37	0.103
PUFA n-6/PUFA n-3	3.68	3.56	0.22	0.623
PUFA/SFA	0.31	0.28	0.04	0.145
UFA/SFA	1.41	1.37	0.04	0.365

^a^ SFA = C4:0 + C6:0 + C8:0 + C10:0 + C11:0 + C12:0 + C13:0 + C14:0 + C15:0 + C16:0 + C17:0 + C18:0 + C20:0 + C21:0 + C22:0 + C23:0 + C24:0. ^b^ UFA = C14:1 + C15:1 + C16:1 + C17:1 + C18:1n-9t + C18:1n-9c + C18:2n-6t + C18:2n-6c + C18:3n-6 + C18:3n-3 + C20:1 + C20:2 + C20:3n-6 + C20:4n-6 + C20:5n-3 + C22:6n-3 + C24:1n-9. ^c^ MUFA = C14:1 + C15:1 + C16:1 + C17:1 + C18:1n-9t + C18:1n-9c + C20:1 + C24:1n-9. ^d^ PUFA = C18:2n-6t + C18:2n-6c + C18:3n-6 + C18:3n-3 + C20:2 + C20:3n-6 + C20:4n-6 + C20:5n-3 + C22:6n-3. ^e^ PUFA n-3 = C18:3n-3 + C20:5n-3 + C22:6n-3. ^f^ PUFA n-6 = C18:2n-6t + C18:2n-6c + C18:3n-6 + C20:2 + C20:3n-6 + C20:4n-6.

## Data Availability

The data will be made available from the corresponding author upon reasonable request.
